# Excessive Refined Carbohydrates and Scarce Micronutrients Intakes Increase Inflammatory Mediators and Insulin Resistance in Prepubertal and Pubertal Obese Children Independently of Obesity

**DOI:** 10.1155/2014/849031

**Published:** 2014-11-16

**Authors:** Mardia López-Alarcón, Otilia Perichart-Perera, Samuel Flores-Huerta, Patricia Inda-Icaza, Maricela Rodríguez-Cruz, Andrea Armenta-Álvarez, María Teresa Bram-Falcón, Marielle Mayorga-Ochoa

**Affiliations:** ^1^Unit of Research in Medical Nutrition, Pediatric Hospital, National Medical Center “Siglo XXI”, Mexican Institute of Social Security, P.O. Box C-029 CSPI, Coahuila No. 5 Col. Roma, 06703 México, DF, Mexico; ^2^Nutrition and Bioprogramming Research Department, National Institute of Perinatology “Isidro Espinosa de los Reyes”, México, DF, Mexico; ^3^Department of Community Health Research, Infantile Hospital of México “Federico Gómez”, México, DF, Mexico; ^4^Faculty of Health Sciences, North University Anahuac, Huixquilucan, MEX, Mexico

## Abstract

*Background.* Low-grade inflammation is the link between obesity and insulin resistance. Because physiologic insulin resistance occurs at puberty, obese pubertal children are at higher risk for insulin resistance. Excessive diets in refined carbohydrates and saturated fats are risk factors for insulin resistance, but calcium, magnesium, vitamin-D, and the omega-3 fatty acids likely protect against inflammation and insulin resistance. *Objective.* To analyze interactions among dietary saturated fat, refined carbohydrates, calcium, magnesium, vitamin D, and omega-3 fatty acids on the risk of inflammation and insulin resistance in a sample of prepubertal and pubertal children. *Methods.* A sample of 229 children from Mexico City was analyzed in a cross-sectional design. Anthropometric measurements, 24 h recall questionnaires, and blood samples were obtained. Serum insulin, glucose, calcium, magnesium, 25-OHD3, C-reactive protein, leptin, adiponectin, and erythrocytes fatty acids were measured. Parametric and nonparametric statistics were used for analysis. *Results.* While mean macronutrients intake was excessive, micronutrients intake was deficient (*P* < 0.01). Inflammation determinants were central obesity and magnesium-deficient diets. Determinants of insulin resistance were carbohydrates intake and circulating magnesium and adiponectin. *Conclusions.* Magnesium-deficient diets are determinants of inflammation, while high intake of refined carbohydrates is a risk factor for insulin resistance, independently of central adiposity.

## 1. Introduction

Pediatric obesity is related to an increased risk of metabolic alterations such as inflammation, insulin resistance, glucose intolerance, and hepatic steatosis, as well as to established pathological conditions such as nonalcoholic fatty liver disease, metabolic syndrome, type 2 diabetes, and cardiovascular disease, either at that time or later in life [1]. Scientific evidence demonstrates that chronic low grade inflammation is the link between obesity and insulin resistance; the main mechanism involved is an increased synthesis of cytokines in adipose tissue and the resident macrophages, which interfere with insulin course and with the expression of genes involved in insulin performance [2]. Moreover, currently it is accepted that inflammation and insulin resistance are the underlying cause of most of the pathological complications and obesity-related comorbidities [3, 4].

Accordingly, it seems clear that inflammation is the route through which obesity results in insulin resistance. However, it is not that simple because in addition to obesity, the etiology of insulin resistance includes genetic and environmental factors. The environmental influence comes mainly from sedentary life styles and dietary factors [5, 6]. Diets in excess of energy and specific nutrients such as too much saturated fat or refined carbohydrate have been implicated in the risk of insulin resistance [7]. However, those dietary disparities do not explain all the cases of insulin resistance suggesting that other dietary factors likely increase the risk to develop insulin resistance. On this regard, diets that are deficient in some particular nutrients such as calcium, magnesium, vitamin D, and the omega-3 polyunsaturated fatty acids have been implicated because they all are involved or influence the metabolic pathways of insulin action. In addition, most of these nutrients have demonstrated anti-inflammatory properties [8–12], which make them potential candidates for insulin resistance management.

The interaction among dietary imbalances on inflammation and insulin resistance has not been explored in pediatric pubertal populations, which is important because children at puberty present certain degree of physiological insulin resistance. Hence, we analyzed the role of dietary macronutrients, as well as saturated fat, calcium, magnesium, vitamin D, and omega-3 fatty acids, on the risk of inflammation and insulin resistance in a sample of prepubertal and pubertal children.

## 2. Methods

### 2.1. Study Population

In a cross-sectional design, a sample of prepubertal and pubertal children was recruited from high schools close to two pediatric hospitals in Mexico City. Selection criteria included body mass index (BMI) above 85th percentile but healthy otherwise, age between 10 and 18 y, and signing informed assent and consent forms by the children and their guardians, respectively, after explaining the objective and characteristics of the study. A group of normal BMI children, relatives of included overweight and obese children, who volunteered to participate, was also analyzed. Children who accepted to participate were asked to attend the Unit of Research in Medical Nutrition in two occasions. The first appointment was scheduled in a weekday to complete a clinical history form, a 24 h recall questionnaire, and to provide a peripheral blood sample. The second appointment was scheduled to obtain a weekend day 24 h recall questionnaire. The study protocol was approved by the Ethics Committee of the Mexican Institute of Social Security (IMSS: R-2010-3603-14) and of the Hospital Infantil de México “Dr. Federico Gómez” (HIM/2011/001; SSa 928).

### 2.2. Procedures

#### 2.2.1. Data Collection

All children and their guardians signed the assent and consent forms. At inclusion, field workers, previously trained and standardized, obtained anthropometric measurements using standard procedures. Weight and height were measured with electronic balances (BWB-700, Tanita Corporation, Tokyo, Japan) and fixed stadiometers (Holtain Limited), respectively. Waist circumference was measured with fiber glass tapes at the midpoint between the iliac crest and the lower rib; the measured waist circumference was compared with the Fernandez reference chart to estimate percentiles [13]. BMI was calculated by dividing weight (kg) by height (m)^2^. BMI* Z-scores* were calculated with the Epi-Info software (EPI-INFO 2000, release 3.2.2).

#### 2.2.2. Dietary Assessment

Diet was assessed using the average of two multiple-pass 24-hour recalls [14]. Previously trained and standardized nutritionists applied in-person recalls to all children using food models and portion estimation tools. Nutrient analysis was performed with the Food Processor software (version 8.0, 2000, ESHA Research Inc., Salem, OR) which includes Mexican foods. Missing foods were added to the database using Mexican Food Exchange System. Macro and micronutrient intakes were assessed according with the recommended dietary intake (RDI) or adequate intake (AI) for age and gender [15, 16]. Food portions were quantified from all food groups included in the diets.

Dietary information is presented as the percentage distribution of macronutrients, as adequacy of recommendations, as each nutrient adjusted by kg body weight, and as quartile of intake. To analyze adequacy of recommendations a diet was considered adequate if it was 100 ± 10% of recommendations, except for energy whose adequacy was 100% and saturated fat whose adequacy was 10% of total energy.

#### 2.2.3. Laboratory Determinations

A peripheral blood sample was obtained after 12 h fasting, collected in free-metal tubes, and centrifuged at 3000 rpm to separate serum, which was preserved at −70°C until laboratory determinations. Circulating glucose, calcium, and magnesium were determined with an enzymatic method (SPIN-React 120, Sant Esteve De Bas, Spain). Insulin (Millipore, Billerica MA, USA) and leptin and adiponectin (Linco Research, Inc., St. Charles, Missouri, USA) were measured by radioimmunoassay using commercial kits. To determine C-reactive protein (DSL UK Ltd, Oxon, UK) ELISA methods were employed, and 25-OHD3 was determined with high performance liquid chromatography [17]. Coefficient of variation was 7–10% for the enzymatic analyses, 10% for insulin and leptin, 7.5% for ELISA assays, and 4% for 25-OHD3.

#### 2.2.4. Erythrocytes Fatty Acids

The omega-3 fatty acid profile was measured in erythrocytes separated from blood samples. Serum was removed immediately after centrifugation and erythrocyte pellets were washed twice with 0.9% saline and stored at −20°C until analysis. Total fat was extracted from 0.5 g of frozen erythrocytes with 4.5 mL of isopropanol; butylated hydroxytoluene was added as an antioxidant (10 ug/mL final volume). Tubes were shaken for 15 min and centrifuged for 5 min at 1,200 ×g at 4°C; the clear supernatant was poured off and dried at 60°C under a stream of nitrogen. Fatty acids were methylated with methanolic HCl 3N. The methyl esters were extracted from the mixture with hexane and analyzed by gas chromatography (Hewlett Packard 5890 series II. Avondale, PA) using a flame ionization detector, and a 100 m × 0,2 mm inside diameter fused silica column coated with 0.2 *μ*m CP Sil 88 (Chrompac, The Netherlands). Fatty acids were identified from chromatograms by comparison with known standards. Fatty acid concentrations were calculated by using a response factor of standard fatty acids. Heptadecanoic acid was added to the samples as an internal standard. Results were expressed as weight percentages of total fatty acids.

#### 2.2.5. Clinical Diagnoses

Children were divided into three groups according to nutritional status using the BMI* Z-score*: normal weight, between −1 and +1 SD; overweight, between +1 and +2 SD; and obese, >2 SD. Children were also stratified by the presence of central obesity using the 90th percentile of waist circumference as cutoff point [13, 18].

To classify children according to insulin resistance status HOMA was calculated using the following formula: (Insulin (*μ*U/mL) ∗ glucose (mmol/L)/22.5); a cutoff point of 3.16 was used to determine insulin resistance [19]. To identify low-grade inflammation we considered a C-reactive protein concentration between 3 and 15 *μ*g [20].

The Tanner stage was established by asking the children to identify themselves in printed pictures representing the different stages. Children were classified as pubertal if the Tanner stage was ≥3 or prepubertal otherwise.

#### 2.2.6. Statistical Analysis

The Minitab statistical package (v.14.2, State College, PA) was used for analysis. A *P* value ≤0.05 was considered for statistical significance. For description, data is presented as mean ± SD and median and range. Student *t*-tests were used to analyze differences by insulin resistance or inflammatory status, as appropriate. 1-sample *t*-tests or 1-sample sign-tests were used to compare actual dietary intakes from recommended dietary intake. Associations of dietary and biochemical alterations, with insulin resistance and inflammation, were analyzed with X^2^ or Fisher test. Partial correlations were used to identify significant predictors of IR and inflammation included predictors were anthropometric, dietary, and circulating factors. For multivariate analyses, logistic regression models, and multiple regression models with the General Linear Model approach were used.

## 3. Results

### 3.1. Sample Characteristics

A sample of 229 children was evaluated. Mean age was 12.17 ± 2.51 y, 53.28% were male, and 64.19% pubertal. The mean BMI* Z-score* of children was 2.10 ± 0.75 SD; 8% percent were classified as normal, 35% as overweight, and 57% as obese. Central obesity was identified in 63.44%, insulin resistance in 82.53%, and inflammation in 37.8% of children.

In average, macronutrients intake was distributed as 50.71 ± 8.67% carbohydrates, 16.8 ± 4.7% protein, and 33.4 ± 7.7% fats; saturated fat intake provided 10.56 ± 3.32% of total energy. The analysis of nutrient intake as percentage of recommendation showed that while the intake of macronutrients was above recommended, the intake of fiber, vitamin D, calcium, and omega-3 fatty acids was below recommendations (Table 1).

Regarding circulating factors, the mean concentrations of insulin and C-reactive protein were above normal values (Table 2) [21, 22]; yet 20% of children presented calcium concentration <10 mg/dL, and 3.8% presented deficiency (<12 ng/mL) and 32.86% insufficiency (between 12 and 20 ng/mL) of 25-OHD3. The fatty acid profile in erythrocytes was available only for a subsample of 90 children; the mean docosahexaenoic acid was below normal (normal values for adolescents = 4.55 ± 0.97 w%) [22].

### 3.2. Partial Correlations

Running partial correlations allowed identifying puberty and serum Mg as the best predictors of waist circumference. In turn, HOMA was positively related with waist circumference but negatively related with adiponectin and serum Mg. Regarding inflammatory mediators, C-reactive protein concentration was positively associated with leptin and negatively associated with adiponectin and dietary calcium. On the other hand, adiponectin correlated negatively with fat intake and waist circumference but positively correlated with circulating calcium and the intake of omega-3 fatty acids.

### 3.3. Diet and Circulating Factors Stratified by Inflammatory and Insulin Resistance Statuses

#### 3.3.1. Inflammatory Status

Children classified as with low-grade inflammation showed higher waist circumference (90.4 ± 13.1 versus 84.3 ± 13.6 cm, *P* = 0.001), BMI* Z-score* (2.37 ± 0.72 versus 1.98 ± 0.78, *P* < 0.001), and borderline HOMA (7.59 ± 4.32 versus 6.63 ± 3.67, *P* = 0.055) than children without inflammation. Also children with inflammation reported lower dietary calcium (16.6 ± 11.2 versus 13.7 ± 7.4 mg/kg, *P* = 0.013) and magnesium (4.13 ± 2.18 versus 5.01 ± 2.6 mg/kg, *P* = 0.006), as well as higher serum leptin concentration (50.75 ± 42.76 versus 38.30 ± 36.47 ng/mL, *P* = 0.022) and borderline lower 25-OHD3 (26.26 ± 9.17 versus 24.16 ± 9.7 ng/mL, *P* = 0.069), than their normal counterparts.

#### 3.3.2. Insulin Resistance Status

Similarly, children classified as with insulin resistance showed greater waist circumference (87.7 ± 13 versus 79.6 ± 11.8, *P* < 0.001) and BMI* Z-score* (2.37 ± 0.72 versus 1.98 ± 0.80, *P* < 0.001), consumed more carbohydrates (200 ± 65 versus 162 ± 54% of recommendation, *P* < 0.001), tended to consume less calcium (15.16 ± 9.5 versus 18.1 ± 11.9 mg/kg, *P* = 0.076) and magnesium (4.53 ± 2.4 versus 5.1 ± 2.5 mg/kg, *P* = 0.094), and presented higher leptin (45.32 ± 39.41 versus 29.82 ± 27.75 ng/mL, *P* = 0.003) and lower adiponectin (9.0 ± 4.79 versus 12.10 ± 4.28 *μ*g/mL, *P* < 0.001) concentrations than their non-insulin resistance counterparts.

### 3.4. Multivariate Analyses of Inflammation and Insulin Resistance, with Dietary and Circulating Predictors

**Figure 1:**
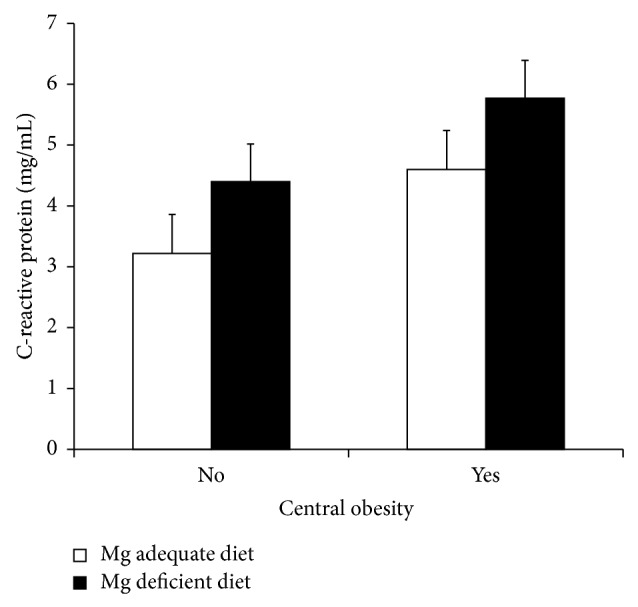
C-reactive protein stratified by central obesity and magnesium deficient diets. Children with central obesity presented higher C-reactive protein than normal waist circumference children (*P* = 0.019). Likewise, children who consumed a magnesium-deficient diet exhibited higher C-reactive protein concentration than children with a normal diet (*P* = 0.028). No interaction between central obesity and deficient diet was detected.

Determinants of inflammation were central obesity (*P* = 0.019) and the intake of a magnesium-deficient diet (*P* = 0.028), after adjusting for the intake of calcium, vitamin D, and omega-3 fatty acids (Figure 1); interaction between central obesity and magnesium-deficient diet was not significant. Because erythrocytes fatty acids were measured only in a subset of children, the analysis was repeated in this group introducing erythrocytes docosahexaenoic acid as a covariate. In this model, the only significant variable was the magnesium-deficient diet (*P* = 0.012); although the erythrocytes docosahexaenoic acid exhibited negative coefficient, this association did not reach statistical significance (coefficient = −0.72 ± 0.51, *P* = 0.160). This result was confirmed by calculating the odds ratio of inflammation as related to erythrocytes docosahexaenoic acid (OR = 0.69; CI_95_ = 0.40, 1.18; *P* = 0.17).

In the case of insulin resistance, the main determinants were central obesity, puberty, fiber, and refined carbohydrates intake, as well as circulating magnesium and adiponectin. While central adiposity, puberty, and carbohydrates intake were risk factors, fiber intake and circulating magnesium, 25-OHD3, and adiponectin were protective (Table 3).

## 4. Discussion

In this cross-sectional study we investigated the interaction among nutrients that are either risky or protective for inflammation and insulin resistance in prepubertal and pubertal children. The most important finding of our study is that children from this population use to consume highly inadequate diets which are excessive or deficient in key nutrients for insulin resistance and inflammation. In fact, the main dietary determinant of inflammation was the intake of a magnesium-deficient diet, while the stronger dietary risk factor for insulin resistance was a high intake of refined carbohydrates. The influence of these dietary factors was independent of central adiposity and puberty.

The association between magnesium intake and inflammation has been extensively reported. Our results are consistent with that information. For instance, Song et al. published a study conducted in a big sample of American women (>11,000); in that study authors detected a significant inverse association between dietary magnesium and C-reactive protein and mentioned that women in the lowest quintile of magnesium intake presented 12% higher C-reactive protein [23]. In our study, the difference in C-reactive concentration between the lowest and the highest quartiles of magnesium intake was 30%, in an even very much small sample size, supporting the plausibility of the association and likely reflecting the high frequency of magnesium-deficient diets consumed by Mexican children. Thus, our result as those of Song et al. confirm the association between inflammation and dietary deficiency of magnesium and suggest that the beneficial effect of magnesium intake on insulin resistance is at least partially mediated by improvement of the low-grade inflammation that accompany obesity.

Regarding macronutrients, we found a strong association between the intake of refined carbohydrates and insulin resistance, consistent with the extensive information in the literature. One of those publications, presented by Bray and Popkin who reviewed several meta-analyses and clinical trials, concludes that refined carbohydrates, mainly those coming from soft drinks, increase the risk of weight gain, fatty liver, and the metabolic syndrome [24], all these are pathological conditions linked to insulin resistance. These authors propose that one of the mechanisms mediating the effect of refined carbohydrates on insulin resistant is through increasing the visceral* de novo* lipogenesis. This statement is supported by our results since we found also insulin resistance was associated with carbohydrates intake and that carbohydrates intake, in turn, was related to waist circumference. Although in our work only refined carbohydrates intake influenced the risk of insulin resistance, it is noteworthy that saturated fat affected negatively the concentration of circulating adiponectin, supporting the reports in the literature that saturated fat intake also increases insulin resistance.

Our study strengths include the concurrent analysis of several positive and negative dietary influences on insulin resistance and on inflammatory mediators, which made it possible to disentangle those factors which are likely confounders. For instance, we did not find any association of dietary or circulating vitamin D on either insulin sensitivity or inflammation, in spite of the high frequency of deficiency (51% of children consumed a vitamin D deficient diet and more than 36% presented circulating 25-OHD3 in the range of insufficiency). Therefore, our results confirm those reported in a meta-analysis that did not find any effect of circulating vitamin D, or supplementation, on metabolic outcomes [24]. Thus, our evidence seems adequate to support the beneficial effect of magnesium on insulin sensitivity and inflammation; such effect seems to be independent of other highly correlated dietary nutrients as calcium, fiber, and the omega-3 fatty acids.

However, several limitations merit consideration, such as the cross-sectional design that does not allow for causal inferences about the role of dietary nutrients on inflammation and insulin resistance. Second, we only applied two multiple-pass 24 h recall to obtain dietary information, and hence measurement errors might have biased the results. In third place, the Tanner stage was not identified by a pediatrician, this could have led to children misclassification; yet we think that the probability is that when identifying themselves in the pictures, children tend to choose a lower Tanner stage than the real one, favoring the results against our hypothesis; this is only speculative though. Lastly, the analysis of tissue omega-3 fatty acids was available only for a small sample, diminishing the power to identify the influence of such important predictor of insulin sensitivity and inflammation. Nevertheless, our results about associations of magnesium and inflammation and refined carbohydrates and insulin resistance are consistent with those in the scientific literature and with the mechanisms observed from experimental studies, improving the plausibility of our results.

Unfortunately we did not find any correlation between dietary and tissue omega-3 fatty acids. Beside mechanistic reasons such as different paths to use or store fatty acids depending on the metabolic status of the children, other possible explanations are insufficient sample size, inadequate information about dietary fatty acids because of only two 24-h recall questionnaires, underestimation of this fatty acids content in Mexican foods, or lack of this information in the Food Processor software. We are working on that, and an investigation regarding this point is underway.

In conclusion, we found that magnesium-deficient diets predict inflammation and that refined carbohydrates predict insulin resistance, independently of central obesity, puberty, and other nutrients deficiencies or excess. The influence of magnesium appears to be through increasing adiponectin secretion, but this hypothesis needs to be proved.

## Figures and Tables

**Table 1:** Nutrient intakes expressed as percentage of recommendation by gender and age.

% of recommendations^a^			
	Median	First, third quartiles	*P* value^b^
Energy	106.45	82.60, 137.40	0.085
Protein	224.51	175.75, 280.79	<0.001
Carbohydrates	190.33	145.36, 233.21	<0.001
Fat	202.78	146.63, 280.07	<0.001
Saturated fat^c^	10.57	8.35, 12.33	0.014
Fiber	59.23	42.94, 78.48	<0.001
Vitamin D	89.40	48.70, 126.1	0.002
Calcium	59.48	41.32, 88.3	<0.001
Magnesium	97.19	73.33, 132.78	0.290
Omega 3	63.00	45.83, 86.65	<0.001

^a^IOM [15, 16]; ^b^sign test for medians was used to assess differences with 100% recommendation for gender and age; ^c^saturated fat is expressed as 10% of total energy.

**Table 2:** Circulating factors as compared to references^1^.

	Mean ± SD	Median	First, third quartiles	Reference
Glucose, mg/dL	89.96 ± 9.06	89.00	85.00, 96	70–99
Insulin, *μ*U/mL	30.68 ± 18.95	26.61	17.70, 38.44	<25.0
Calcium, mg/dL	10.41 ± 0.63	10.35	10.0, 10.7	9.5–10.5
Magnesium, mg/dL	2.26 ± 0.22	2.1	2.0, 2.3	1–4.9
25-OHD3, ng/mL	25.04 ± 9.47	23.15	17.81, 31.10	20–100
Docosahexaenoic acid, w/%	3.30 ± 0.94	3.31	2.74, 3.90	>4.00
Eicosapentaenoic acid, w/%	0.37 ± 0.21	0.34	0.20, 0.50	>0.35
Linolenic acid, w/%	0.28 ± 0.15	0.25	0.20, 0.30	>0.14
Leptin, ng	42.52 ± 37.99	30.14	19.32, 48.79	—
Adiponectin, *μ*g	9.59 ± 4.84	9.23	4.58, 12.91	—
C-reactive protein, mg	3.52 ± 4.40	2.04	0.58, 4.90	<3.00

^1^ *n* = 229, except for omega-3 fatty acids, where *n* = 90.

**Table 3:** Determinants of insulin resistance measured as HOMA >3.16^*^.

	Quartile		OR	CI_95_	*P*-value
Protein	1	≤175.75			
	2	175.76–224.51	0.84	0.28, 2.55	0.760
	3	224.52–280.79	1.07	0.32, 3.67	0.908
	4	≥280.80	1.04	0.23, 4.79	0.956

Carbohydrates	1	≤145.36			
	2	145.37–190.33	3.52	1.07, 11.50	0.038
	3	190.34–233.21	25.45	5.28, 122.61	<0.001
	4	≥233.22	21.68	4.25, 110.66	<0.001

Lipids	1	≤146.63			
	2	146.64–207.78	0.47	0.11, 1.96	0.302
	3	207.79–280.07	0.14	0.02, 0.98	0.048
	4	≥280.08	0.26	0.03, 2.64	0.256

Fiber	1	≤42.94			
	2	42.95–59.23	0.21	0.06, 0.72	0.013
	3	59.24–78.48	0.49	0.13, 1.83	0.289
	4	≥78.49	0.17	0.04, 0.73	0.017

Saturated fat	1	≤15.88			
	2	15.89–22.44	1.30	0.33, 5.08	0.708
	3	22.45–31.53	1.56	0.27, 9.03	0.621
	4	≥31.54	2.35	0.28, 19.49	0.429

Serum Ca			1.42	0.70, 2.87	0.329
Serum Mg			0.05	0.01, 0.51	0.011
25-OHD3			0.98	0.91, 1.04	0.468
Leptin			1.03	0.98, 1.09	0.238
Adiponectin			0.01	0.00, 0.62	0.030
Central obesity			3.46	1.43, 8.36	0.006
Puberty			3.12	1.20, 8.12	0.020

^*^Logistic regression analysis. Control group for dietary factors is quartile 1. Circulating factors were introduced as continuous variables.
